# Dual-scale TiO_2_ and SiO_2_ particles in combination with a fluoroalkylsilane and polydimethylsiloxane superhydrophobic/superoleophilic coating for efficient solvent–water separation†

**DOI:** 10.1039/c9ra02700a

**Published:** 2019-06-28

**Authors:** Frances L. Heale, Maud Einhorn, Kristopher Page, Ivan P. Parkin, Claire J. Carmalt

**Affiliations:** Department of Chemistry, University College London 20 Gordon Street London WC1H 0AJ UK c.j.carmalt@ucl.ac.uk +44 (0)20 7679 7528

## Abstract

Surfaces that have unique wettabilities and are simultaneously superhydrophobic with water contact angles > 150°, and superoleophilic with oil contact angles < 5°, are of critical importance in the oil/solvent–water separation field. This work details the facile preparation of highly efficient oil–water separation devices that successfully combine hierarchical surface roughening particles and low surface energy components with porous substrates. Coatings were generated using TiO_2_ and hydrophobic-SiO_2_ micro/nanoparticle loadings which were then embedded within polydimethylsiloxane, commercially known as Sylgard® 184, and 1*H*,1*H*,2*H*,2*H*-perfluorooctyltriethoxysilane (FAS) polymer mixtures. The resulting slurries were dip coated onto copper meshes with varying pore diameters (30, 60 and 100 meshes had 595, 250 and 149 μm pore dimensions respectively). Functional testing proved that mesh substrates coated in the lowest Sylgard® 184 : FAS polymer ratio formulations displayed heightened water repellency and retained their superoleophilic properties upon repeat testing. The largest average water contact angle of 145 ± 1°, was recorded on a copper 30 mesh substrate with a coating comprising H-SiO_2_ microparticles and TiO_2_ nanoparticles in a 1 : 9 polymer mixture of Sylgard® and FAS. The coating's extreme oil affinity was supported by high solvent–water separation efficiencies (≥99%) which withstood numerous testing/washing cycles.

## Abstract 2

Surfaces that have unique wettabilities and are simultaneously superhydrophobic with water contact angles > 150°, and superoleophilic with oil contact angles < 5°, are of critical importance in the oil/solvent–water separation field.

## Graphical abstract

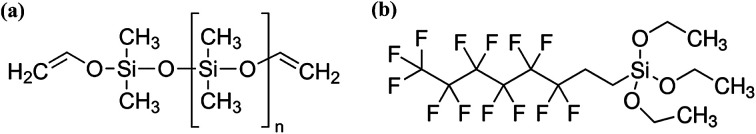

## 1. Introduction

The increasing volume of oil and/or solvent contamination in industrial wastewater is a lasting contribution to the deterioration of our marine and terrestrial ecosystems. The food, leather, textile and petrochemical industries all generate significant amounts of unwanted oily water during daily production.^1^ When these continuous waste outputs couple with freak accidents, such as oil spills, the natural environment suffers severe implications for up to one decade after each isolated event.^2^ During the period between 2010 and 2017 there was more than 47 000 tonnes of petrochemical oil spilt in aquatic environments, 80% of this amount was lost in just ten incidents.^3^ It is therefore of great importance to engineer efficient oil–water separation techniques capable of rapidly cleaning the environment in the event of future spills and to decontaminate oily industrial waste.

Interactions between liquids and surfaces are central to this field of research. Superhydrophobic materials often make use of nano and/or microscale protrusions combined with very low surface energy modifications to achieve static water contact angles ≥ 150°.^4,5^ Water droplets upon these materials can exist in a Wenzel state, whereby the droplets ‘stick’ to all parts of the surface, or Cassie–Baxter regime where droplets ‘slip’ on a lubricating air layer trapped within surface asperities.^6,7^ The literature details various ways of fabricating novel water repellent surfaces inspired by nature's waterproof materials such as lotus leaves butterfly wings and geckos' feet.^5^ Plasma enhanced chemical vapor deposition (PECVD) was carried out by Bico *et al.* to generate carbon flake-like multiscale surface structures.^8^ Aerosol-assisted chemical vapour deposition (AACVD) of Sylgard® 184 or polydimethylsiloxane (PDMS) and tetraethyl orthosilicate (TEOS) afforded appropriately rough and transparent coatings on glass substrates.^9–11^ Alternative superhydrophobic generating methods such as chemical etching,^12^ electrospinning^13,14^ and lithographic imprinting/templatation^15^ have enhanced the functionality of oil–water separation, self-cleaning, anti-icing and anti-corrosion materials.^12,16–18^

Whilst water repellency is crucial to certain oil–water separation devices, materials with two wettability regimes have been known to improve separation efficiency. A surface displaying both superhydrophobic ‘water-hating’ and superoleophilic ‘oil-loving’ properties can be tuned for selective separation providing its surface energy resides between that of the solvent/oil and water it aims to separate (approximately between 30 mN m^−1^ and 70 mN m^−1^).^19^ Filtration and/or absorption are successful types of oil-removing techniques that encompass both size exclusion and wettability selectivity mechanisms. Pore sizes are commonly designed to either let oil run straight through the mesh/membrane or for the material to absorb the hydrocarbon whilst water is repelled.^20^

Copper meshes have been etched with nitric acid and alkali (NaOH/K_2_S_2_O_8_) solutions before modification with self-assembled monolayers of hexadecanethiol and dodecanethiol respectively. High water contact angles, ∼150°, and low oil contact angles, <5°, produced materials that separated at least 97% of water from an oily solution.^21,22^ Vertically-aligned multi-walled carbon nanotubes were synthesised on a stainless steel mesh *via* CVD. Needle-likes tubes created a high surface area and aided capillary action; the oil contact angle was recorded at 0° and oil penetrated the mesh in 0.4 s.^23^ Li *et al.* fabricated candle soot and silica coated meshes that repelled hot water and corrosive liquids. These coated meshes worked as effective gravity separation devices with ∼99% of organic solvents permeating through the device.^24^ Nano protrusions have also been introduced by electrochemical etching, a copper mesh was anodized in a NaOH solution (1 M) to create a needle-like Cu(OH)_2_ film. The roughened substrate was then coated in a self-assembled monolayer of 1*H*,1*H*,2*H*,2*H*-perfluorooctyltriethoxysilane (FAS) to form a durable solvent/water separator.^25^ Spin coating metal mesh substrates with ZnO nanorods has been yet another effective fabrication method.^26^

Swapping a porous substrate for porous structures (on special materials) enhances robustness by removing the possibility for coating stripping. Tu *et al.* designed a micro-bead and nanofiber film created in one step by spraying onto any given substrate. The water contact angle on these surfaces was not as high as other but the cost and fabrication time were drastically reduced.^27^ A thermoplastic polyurethane mat, created *via* electrospinning, immersed in a hexadecyltrimethoxysilane (HDTMS) modified nanosilica solution also generated an efficient structured separation material.^28^ Several other surface modification methods such as electrostatic deposition, grafting polymerisation, spray drying and photo-initiated polymerisation have gained traction by utilising various polymers, gels and biomaterials.^29,30^

Herein we present an extremely facile one-pot method to fabricate highly efficient oil–water separation devices, >99% efficiency. Combinations of hierarchical surface roughening particles and low surface energy components upon porous substrates lead to enhanced robustness and functionality when tested with a variety of common industrial solvents; this elevated device effectiveness across a substrate and solvent range has substantially advanced work in this area. Here, TiO_2_ and hydrophobic-SiO_2_ micro/nanoparticles were embedded within polydimethylsiloxane (Sylgard® 184) and 1*H*,1*H*,2*H*,2*H*-perfluorooctyltriethoxysilane (FAS) polymer mixtures. Resulting slurries were dip coated onto copper meshes with varying pore diameters (30, 60 and 100 meshes had 595, 250 and 149 μm pore dimensions respectively). Water contact angles, solvent separation efficiencies and mechanical stability were found to outperform many of the existing optimum superhydrophobic/superoleophilic filtration surface previously documented.

## 2. Experimental

### 2.1. Materials

Anatase TiO_2_ particles (60–200 nm diameter), SiO_2_ particles (0.5–1.0 μm diameter and 5–15 μm diameter), 1*H*,1*H*,2*H*,2*H*-perfluorooctyltriethoxysilane, ethanol (reagent grade), toluene (reagent grade), hexane (reagent grade) and dichloromethane (reagent grade) were purchased from Sigma Aldrich. P25 TiO_2_ nanoparticles (21 nm diameter) were acquired from Degussa. Copper mesh substrates (30, 60 and 100 meshes had 595, 250 and 149 μm pore dimensions respectively) were bought from Alfa Aesar and Sylgard® 184 and was acquired from Dow Corning.

### 2.2. Preparation of polymer stock solutions

**Fig. 1:**
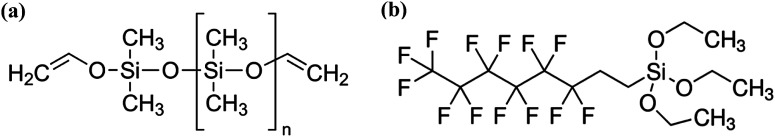
Structure of (a) Sylgard® 184 polymer and (b) 1*H*,1*H*,2*H*,2*H*-perfluorooctyltriethoxysilane prior to polymerisation.

Sylgard® 184 curing agent and elastomer base in a 1 : 10 mass ratio (30.00 g combined mass) were dissolved in ethanol (150.00 g), Fig. 1a. The polydimethylsiloxane polymer was magnetically stirred for 2 hours. A colourless, viscous mixture was achieved.

A 1*H*,1*H*,2*H*,2*H*-perfluorooctyltriethoxysilane (FAS) solution was prepared by magnetically stirring FAS (1.00 g) in ethanol (99.00 g) for 2 hours to produce a clear/colourless solution, Fig. 1b.

### 2.3. Pre-functionalisation of SiO_2_ particles (H-SiO_2_)

Due to the innate hydrophilicity of unrefined silica, 0.5–1.0 μm diameter SiO_2_ particles (5.00 g) and 5–15 μm diameter SiO_2_ particles were sonicated (40 kHz) for 30 min in the fluoroalkylsilane (FAS) stock solution (25 mL). The cloudy mixture was oven dried at 60 °C for 2 hours to allow for complete solvent evaporation, a fine white hydrophobic-SiO_2_ (H-SiO_2_) powder remained. The TiO_2_ particles did not require this prefunctionalisation step.

### 2.4. Generation of functional oil–water separation meshes

Optimised mineral systems (dual-scale TiO_2_ particles, dual-scale hydrophobic-SiO_2_ (H-SiO_2_) particles or mixed-scale H-SiO_2_ particles with TiO_2_ particles) were placed into separate centrifuge tubes and combined with the Sylgard® 184 and 1*H*,1*H*,2*H*,2*H*-perfluorooctyltriethoxysilane (FAS) stock solutions, Table S1.† All mixtures were sonicated (40 kHz) for 30 min to form uniform suspensions. Copper 30, 60 and 100 mesh substrates (15 × 15 cm) were loosely rolled and immersed in the suspensions for 2 min (one 30, 60 and 100 mesh per coating mixture) prior to oven drying at 60 °C for 2 hours.

For ease of reference the following particle combinations and loadings (embedded in a polymer mixture) have been abbreviated, Table 1. Optimised functional coatings have also been labelled, Table 2.

**Table 1:** Abbreviated labelling for particle combinations and loadings within oil–water separation coatings. Hydrophobic-SiO_2_ (H-SiO_2_) particles were generated by functionalising the as received SiO_2_ mineral (5.00 g) in a 1*H*,1*H*,2*H*,2*H*-perfluorooctyltriethoxysilane (FAS) (1.00 g)/ethanol (99.00 g) mixture

Coating label	Particle loading/g		
	TiO_2_		H-SiO_2_ 5–15 μm
	21 nm	60–200 nm	
A	0.6	—	0.6
B	0.6	—	0.6
C	1.5	1.5	—

**Table 2:** Abbreviated labelling for particle combinations and loadings within oil–water separation coatings. Hydrophobic-SiO_2_ (H-SiO_2_) particles were generated by functionalising the as received SiO_2_ mineral (5.00 g) in a 1*H*,1*H*,2*H*,2*H*-perfluorooctyltriethoxysilane (FAS) (1.00 g)/ethanol (99.00 g) mixture

Coating label	Particle loading/g			Mixture of polymer stock solutions/g		Sylgard® 184 : FAS ratio
	TiO_2_		H-SiO_2_ 5–15 μm	Sylgard® 184	FAS	
	21 nm	60–200 nm				
D	0.6	—	0.6	3	27	1 : 9
E	0.6	—	0.6	30	—	0 : 1
F	1.5	1.5	—	6	24	1 : 4
G	0.6	—	0.6	6	24	1 : 4

### 2.5. Characterisation

X-ray photoelectron spectroscopy (XPS) was performed on a Thermo Scientific XPS K-Alpha X-ray Photoelectron Spectrometer with a monochromated AL Kα X-ray source at 1486.6 eV. Fourier transform infrared (FT-IR) spectroscopy was carried out using Bruker Alpha Platinum-ATR equipment (650 to 4000 cm^−1^). Surface morphologies were investigated using a JEOL JSM-6301F scanning electron microscope (SEM) with an acceleration voltage of 5 or 10 kV.

### 2.6. Functional testing

Three water contact angles were measured per coating at ambient temperature *via* the sessile-drop method using a FTA 1000 optical contact angle meter (5 μL water droplet). An average value and associated error were calculated for each sample. The tilting angle, defined as the angle at which a water droplet readily slides off a slanted surface (fixed droplet volume of 0.5 mL), was recorded using a digital angle finder. Averages and standard deviations were calculated.

**Fig. 2:**
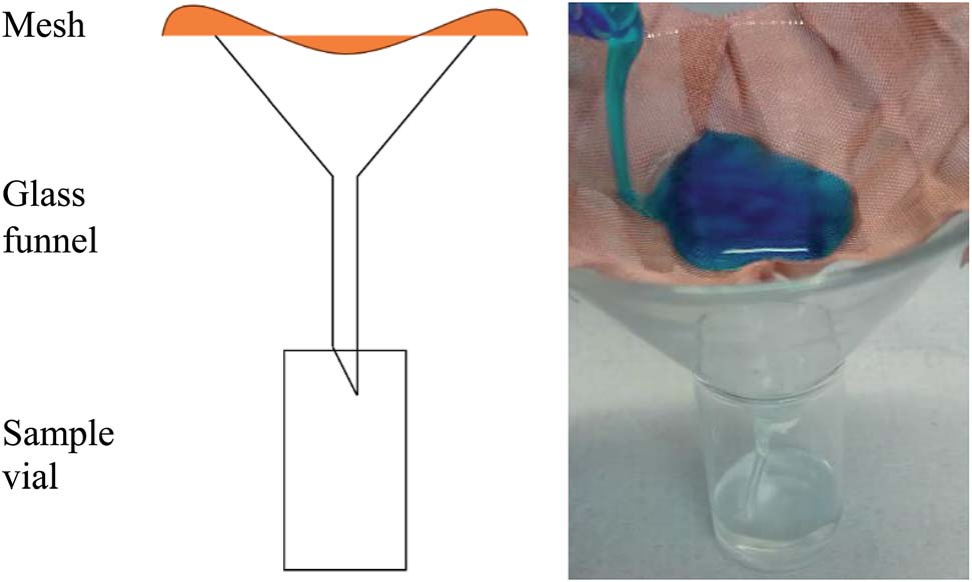
Schematic and image of solvent–water separation efficiency apparatus. Mixtures of known compositions were poured onto various coated sample meshes. The mass of solvent collected after 30 s of separation provided relevant information for the calculation of a comparable separation efficiency percentage.

According to the experimental setup in Fig. 2, solvent–water separation efficiencies were carried out by rapidly pouring oily mixtures over the selected separation device and recording the mass of solvent collected after 30 s (later converted to a percentage of the total solvent content to be separated). Three repeat readings were recorded per separation solution and the initial solvent mass present in the 10 mL mixture was known. One part solvent three parts water (25%), solvent and water in equal parts (50%) and three parts solvent one part water (75%) mixtures of toluene–methylene blue water, hexane–methylene blue water and dichloromethane–methylene blue water (total volume, 10 mL) were the chosen testing solutions.

Meshes were washed with ethanol (5 mL) before and after same solvent separations and new functional meshes were used when solvents were interchanged. Methylene blue water was utilised to prove no water had permeated through the separation mesh as the blue coloured water was easily distinguished from the colourless solvent. Sample durability was further assessed using the Scotch tape test and acid and base baths: Scotch tape was firmly applied to cover the whole sample surface and then ripped/removed at speed 3 times.

## 3. Results and discussion

A one-pot synthesis was used to generate a range of highly functional ‘water hating’ and ‘oil loving’ coatings for application in oil–water separation. Various iterations of TiO_2_ and hydrophobic-SiO_2_ (H-SiO_2_) micro/nanoparticles were dispersed in Sylgard® 184 and 1*H*,1*H*,2*H*,2*H*-perfluorooctyltriethoxysilane (FAS) mixtures, as detailed in Tables S1,†1 and 2. The resulting coatings were dip coated onto 3 types of copper meshes (30, 60 and 100 meshes had 595, 250 and 149 μm pore dimensions respectively).

### 3.1. Characterisation

**Fig. 3:**
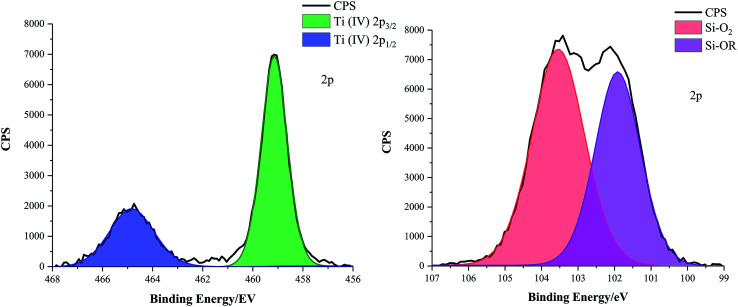
Deconvoluted X-ray photoelectron spectroscopy (XPS) Ti 2p and Si 2p scans of coating D comprising SiO_2_ 5–15 μm particles (0.6 g) and TiO_2_ 21 nm particles (0.6 g) in a 1 : 9 polymer mixture of Sylgard® 184 and FAS.

X-ray photoelectron spectroscopy (XPS) was carried out on the functional coatings to identify the oxidation state of Ti and chemical environments surrounding Si atoms, Fig. 3. Elemental scans of the dried optimised TiO_2_ particle containing coatings (D, E, F and G (formulations listed in Table 2)) confirmed that Ti was constantly in the +4 oxidation state; a 2p doublet was characteristic of the 459.00 eV Ti 2p_3/2_ and the 464.70 eV Ti 2p_1/2_ peaks. Additionally, optimised coating formulations D, E and G also contained SiO_2_ particles. For sample D, consisting of SiO_2_ 5–15 μm particles (0.6 g) and TiO_2_ 21 nm particles (0.6 g) in a 1 : 9 polymer mixture of Sylgard® 184 and FAS, the Si 2p peak was deconvoluted into a 104.00 eV Si–O_2_ and a 101.82 eV Si–OR environment. Values agreed with the literature and were consistent with other TiO_2_ and SiO_2_ particle containing samples.^31–34^

The X-ray diffraction pattern of coating D displays a high intensity TiO_2_ anatase peak at 25.4° and subsequently less intense peaks at 36.7°, 37.8°, 38.8°, 47.9°, 54.6°, 55.1° and 62.5°. A medium intensity rutile peak was identified at 27.4° and SiO_2_ particles were attributed to the quartz peak at 20.8°. As coating F was absent of SiO_2_ particles the only detectable peaks were characteristic of the anatase and rutile phases of TiO_2_. All coating patterns presented in Fig. S1† agreed with particle standards and the Inorganic Crystal Structure Database.

**Fig. 4:**
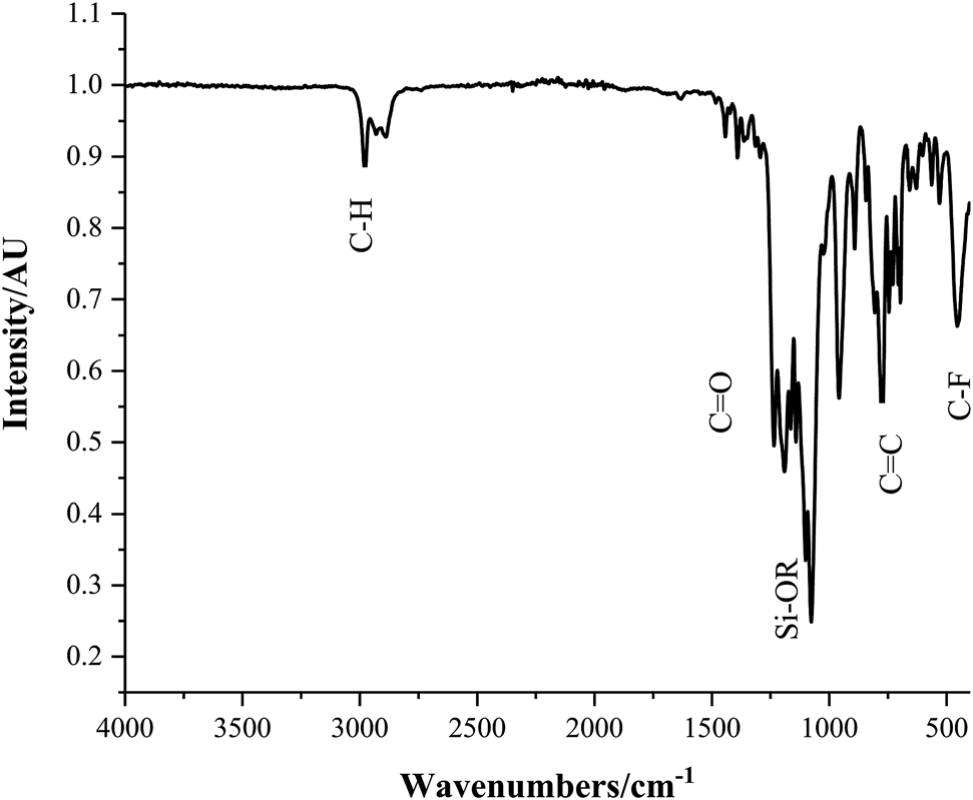
Fourier transform infrared (FT-IR) spectrum of coating D; SiO_2_ 5–15 μm particles (0.6 g) and TiO_2_ 21 nm particles (0.6 g) in a 1 : 9 polymer mixture of Sylgard® 184 and FAS.

Fourier transform infrared (FT-IR) analysis, consistent with the NIST Standard Reference Database, resulted in the identification of several bending and stretching modes characteristic of the Sylgard® 184 and 1*H*,1*H*,2*H*,2*H*-perfluorooctyltriethoxysilane (FAS) polymer mixtures, Fig. 4. Samples containing hydrophobic-SiO_2_ (H-SiO_2_) and/or TiO_2_ particles embedded in 1 : 0, 0 : 1, 1 : 1, 1 : 4 and 1 : 9 Sylgard® 184 : FAS ratios all had similar polymer transmittance bands. A sharp peak at 3120 cm^−1^ was seen in the spectra of coatings D, E, F and G which depicted the C–H alkane stretch present in both polymers. Varying intensities of the following peaks were observed: 1257 cm^−1^ (sh, w) intense out of phase vinyl ether stretch in Sylgard® 184,^35^ 1010 cm^−1^ (s) Si–OR Sylgard® 184 and FAS stretch, 790 cm^−1^ (m) alkene out of plane bending in Sylgard® 184, 622 cm^−1^ (w) C–F FAS stretch and 457 cm^−1^ (w) antisymmetric Si–(CH_3_) Sylgard® 184 stretch.^35^ The presence of these C–F FAS stretches were synonymous with functional effectiveness; fluorine's extreme electronegativity made it only weakly susceptible to fleeting dipoles that form the basis of van der Waals forces. Consequently, the samples' fluorinated carbon chains had small intermolecular forces and therefore the low surface energy hydrophobic requirement.

Scanning electron microscopy (SEM) images provided visual information about the topography of separation materials, particle size/distribution and the extent of copper mesh substrate pore blockage. This qualitative evidence was attributed to water contact angle measurements as well as separation efficiency results. Initial trial coatings containing TiO_2_ particles in high ratios of Sylgard® 184 to FAS (1 : 1 or 1 : 0) resulted in almost complete pore blockage, Fig. S2.† As expected, pore obstruction physically impeded the passage of any solvent through the membrane and resulted in poor separation efficiencies < 25%. Coatings with higher proportions of the more viscous Sylgard® 184 polymer were also seen to reduce the prominence of particle roughening structures thus had reduced average water contact angle values, <110°.

**Fig. 5:**
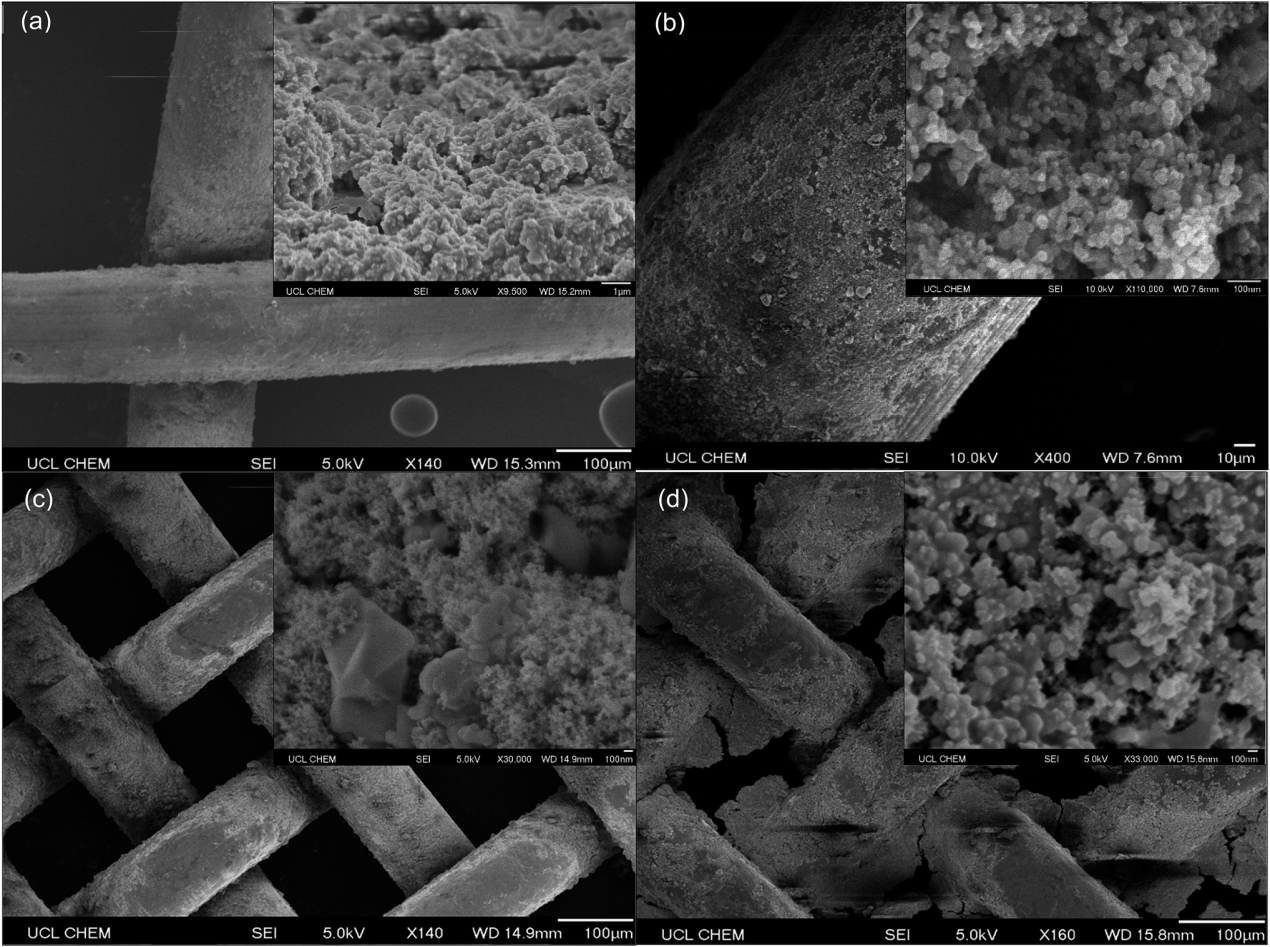
SEM images of (a) coating D on a copper 30 mesh substrate (SiO_2_ 5–15 μm particles (0.6 g) with TiO_2_ 21 nm particles (0.6 g) in a 1 : 9 polymer mixture of Sylgard® 184 and FAS), (b) coating D on a copper 60 mesh substrate (SiO_2_ 5–15 μm particles (0.6 g) with TiO_2_ 21 nm particles (0.6 g) in a 1 : 9 polymer mixture of Sylgard® 184 and FAS), (c) coating E on a copper 100 mesh substrate (SiO_2_ 5–15 μm particles (0.6 g) with TiO_2_ 21 nm particles (0.6 g) in a 0 : 1 polymer mixture of Sylgard® 184 and FAS) and (d) coating F on a copper 100 mesh substrate (TiO_2_ 60–200 nm particles (1.5 g) with TiO_2_ 21 nm particles (1.5 g) in a 1 : 4 polymer mixture of Sylgard® 184 and FAS).

Fig. 5a presents SEM images of the unobstructed pores of a 30 mesh (595 μm pore dimension) copper substrate functionalised with coating D (SiO_2_ 5–15 μm particles (0.6 g) with TiO_2_ 21 nm particles (0.6 g) in a 1 : 9 polymer mixture of Sylgard® 184 and FAS). Dual scale surface structures were accentuated in this coating due to the reduced quantity of Sylgard® 184 and were reflected in the high average water contact angle, 145 ± 1°. However, solvent–water separation testing indicated that the pores were too large to selectively filter solvents from water and so the use of copper 30 mesh substrates were discontinued. Fig. 5b displays a section of coating D adhered to a copper 60 mesh substrate (250 μm pore dimension). Once again, imaging proved that pore openings were clear. The average water contact angle remained relatively high, ∼135°, and the toluene–water separation efficiency in equal parts was recorded as 100%. Fig. 5c shows images of coating E (SiO_2_ 5–15 μm particles (0.6 g) with TiO_2_ 21 nm particles (0.6 g) in a 0 : 1 polymer mixture of Sylgard® 184 and FAS) on copper 100 mesh substrates (149 μm pore dimensions). Open pore structures were conserved, due to the absence of Sylgard® 184, and there was a clear presence of protruding micro and nanoscale particles. The associated average water contact angle and toluene–water separation efficiency in equal parts were 147 ± 1° and 99 ± 1% respectively. Lastly, Fig. 5d displays coating F on a copper 100 mesh substrate (TiO_2_ 60–200 nm particles (1.5 g) with TiO_2_ 21 nm particles (1.5 g) in a 1 : 4 polymer mixture of Sylgard® 184 and FAS). The small pores were ∼50% blocked by this polymer mixture making this an unsuitable solvent–water separation device despite preserving dual scale surface contours, average water contact angle of 146 ± 1°. SEM analysis lead to the assumption that coatings with high proportions of the non-viscous fluorinated FAS polymer mixture created highly structured surface topographies and maximised the appropriate pore area of copper 60 mesh (and in some cases copper 100 mesh) substrates. This in turn elevated the average contact angle values and enhanced efficiency of solvent separation; low Sylgard® 184 levels were tolerated to preserve device coating robustness.

### 3.2. Functional testing

**Fig. 6:**
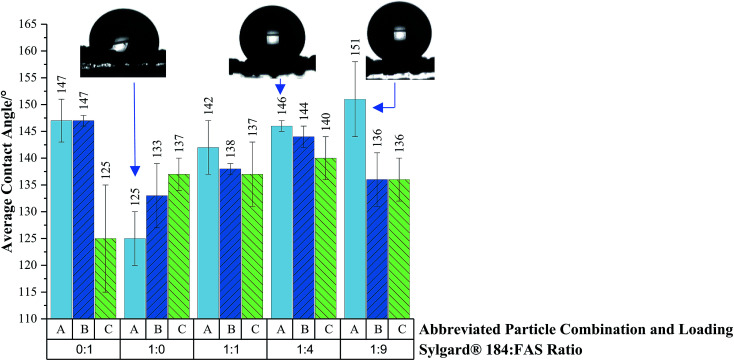
Average water contact angle data on coatings containing various particle combinations and loadings embedded in a Sylgard® 184 and FAS polymer mixture. Abbreviated particle combination and loadings are as follows; A – TiO_2_ 60–200 nm particles (0.6 g) with TiO_2_ 21 nm particles (0.6 g), B – SiO_2_ 5–15 μm particles (0.6 g) with TiO_2_ 21 nm particles (0.6 g) and C – TiO_2_ 60–200 nm particles (0.6 g) with TiO_2_ 21 nm particles (0.6 g). Each particle combination was embedded in 0 : 1, 1 : 0, 1 : 1, 1 : 4 and 1 : 9 Sylgard 184 : FAS ratios. Coatings were applied to copper 60 mesh substrates prior to oven drying. Error bars show the maximum and minimum values obtained after three repeat readings.

Average water contact angle results are plotted in Fig. 6 to demonstrate the impact of changing particle loading/combination and polymer mixture on hydrophobicity. The highly durable, viscous and adhesive Sylgard® 184 polymer had to be delicately balanced with the easily abraded, non-viscous and highly water repellent fluoroalkylsilane (FAS) polymer to achieve a formulation that wouldn't compromise membrane pore size (for effective solvent–water separation) but maintain functionality and durability. Coatings were prepared with 1 : 0, 0 : 1, 1 : 1, 1 : 4 and 1 : 9 Sylgard® 184 : FAS polymer ratios with embedded hydrophobic-SiO_2_ (H-SiO_2_) and/or TiO_2_ loading combinations and functionally contrasted. With the majority of polymer combinations dual scale H-SiO_2_ particles produced the lowest average water contact angle values, for example 125 ± 10° was obtained with the 0 : 1 Sylgard® 184 : FAS polymer ratio mixture. Dual scale TiO_2_ particles consistently generated the highest average water contact angle results for each coating composition (with the exception of the Sylgard® 184 : FAS ratio of 1 : 0), a maximum of 151 ± 7° was achieved with the 1 : 9 Sylgard® 184 : FAS polymer mixture. The coatings that contained the highest concentration of Sylgard® 184 often performed comparatively worse than mesh coatings with a larger FAS content. These findings were expected, the abundance of –C–F polymer bonds in FAS is known to effectively reduce a material's surface energy and consequently increase the average water contact angle value. All samples were in the Cassie–Baxter regime, assumed from low tilting angle results, and improved upon the hydrophobicity of separation meshes detailed in the literature. Fore example. Xue's group generated a superhydrophilic mesh whilst Zhang's coated mesh afforded average water contact angles < 130°.^36,37^

**Fig. 7:**
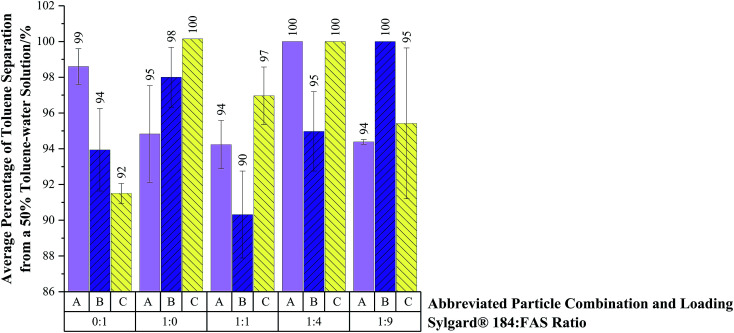
Average percentage of toluene separated from a 50% toluene–water solution on coatings containing various particle combinations and loadings embedded in a Sylgard® 184 and FAS polymer mixture. Abbreviated particle combination and loadings are as follows; A – TiO_2_ 60–200 nm particles (0.6 g) with TiO_2_ 21 nm particles (0.6 g), B – SiO_2_ 5–15 μm particles (0.6 g) with TiO_2_ 21 nm particles (0.6 g) and C – TiO_2_ 60–200 nm particles (0.6 g) with TiO_2_ 21 nm particles (0.6 g). Each particle combination was embedded in 0 : 1, 1 : 0, 1 : 1, 1 : 4 and 1 : 9 Sylgard 184 : FAS ratios. Coatings were applied to copper 60 mesh substrates prior to oven drying. Error bars show the maximum and minimum values obtained after three repeat readings.

Average percentage of toluene separated from 25%, 50% and 75% toluene solutions (75%, 50% and 25% water respectively) were contrasted on coated 60 mesh copper substrates, *via* the setup in Fig. S3.† The H-SiO_2_ and TiO_2_ particle combination, B, embedded in a 1 to 9 Sylgard® 184 : FAS mixture was the most effective at separating 25% toluene–50% water solutions on copper 60 mesh substrates (95 ± 6% separation efficiency). Fig. 7 illustrates that all H-SiO_2_/TiO_2_ particle combinations, A, B and C, reached 100% separation efficiencies for 50% toluene–50% water solutions when coupled with their optimised Sylgard® 184 : FAS polymer mixture. Subsequently, high separation efficiencies of 99 ± 1% were achieved on dual scale H-SiO_2_ as well as H-SiO_2_/TiO_2_ containing particle systems embedded in a 1 to 9 Sylgard® 184 : FAS mixture when tested with 75% toluene–25% water solutions. All particle and polymer mixture combinations had slightly reduced separation efficiencies for 25% toluene–75% water solutions but remained >90%.

Final separation efficiencies on optimised coatings on copper 60 and 100 separation meshes have been recorded in Table S2.† Coating D (SiO_2_ 5–15 μm particles (0.6 g) with TiO_2_ 21 nm particles (0.6 g) in a 1 : 9 polymer mixture of Sylgard® 184 and FAS) coating E (SiO_2_ 5–15 μm particles (0.6 g) with TiO_2_ 21 nm particles (0.6 g) in a 0 : 1 polymer mixture of Sylgard® 184 and FAS), coating F (TiO_2_ 60–200 nm particles (1.5 g) with TiO_2_ 21 nm particles (1.5 g) in a 1 : 4 polymer mixture of Sylgard® 184 and FAS) and coating G (SiO_2_ 5–15 μm particles (0.6 g) with TiO_2_ 21 nm particles (0.6 g) in a 1 : 4 polymer mixture of Sylgard® 184 and FAS) were functionally tested using various solvent systems. Table S2† documents solvent separation efficiencies for toluene–, hexane– and dichloromethane–water solutions along with associated errors.

The results indicated that coated copper 60 mesh substrates again were most likely to generate a favourable separation efficiency when compared to coated copper 100 mesh substrates. The larger pore diameter remained unblocked after coating with even the most viscous of polymer mixtures and therefore allowed toluene, hexane and dichloromethane to filter through the mesh; small pore 100 mesh substrate blockages most strongly inhibited the densest solvent, dichloromethane, from filtering through the device and consequently has not been featured in Table S2.† Despite the exceptional performance of all optimised coatings D–G, formulation F on 60 mesh substrates was an extremely effective separation coating with 100 ± 0%, 85 ± 0% and 97 ± 0% efficiencies for toluene–, hexane– and dichloromethane–water solutions respectively. The innately hydrophobic dual scale TiO_2_ particles embedded coating F's 1 : 4 Sylgard® 184 : FAS mixture helped elevate the average water contact angle, 146 ± 1°. This favourable particle combination coupled with the relatively high proportion of FAS in the coating elevated average contact angles and improved separation potential. Various other separation materials that used longer more elaborate fabrication procedures have been well documented over recent years. Separation efficiencies ∼ 95% were achieved but the vast number of readily available oils/solvents and the absence of a standardized separation method makes it difficult to directly contrast like results from across the field.^36–41^

**Fig. 8:**
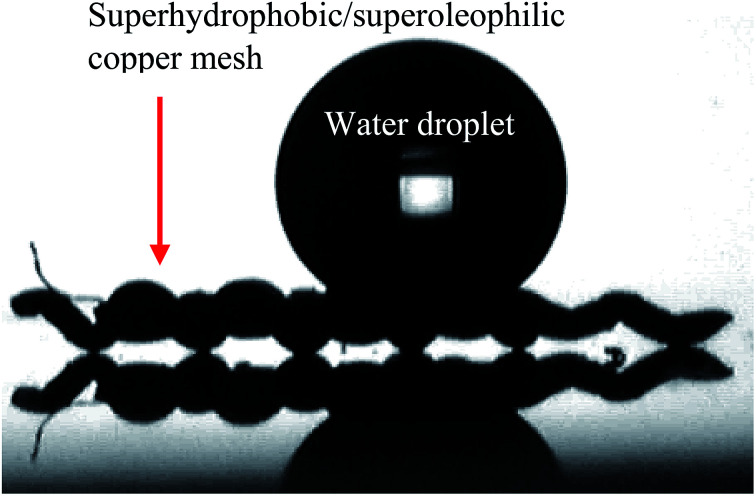
Water droplet on a copper 60 mesh substrate with a coating D surface (SiO_2_ 5–15 μm particles (0.6 g) with TiO_2_ 21 nm particles (0.6 g) in a 1 : 9 polymer mixture of Sylgard® 184 and FAS). Image was recorded post separation functional testing.

Due to the adhesive properties of Sylgard® 184, optimised coatings D, F and G remained comparably functional, within the original error limits represented in Fig. 6, after 3 wash–separation–wash cycles and the Scotch tape test. Fig. 8 displays the water contact angle image post separation testing of coating D; unaltered water repellency further supports coating robustness. The 1 : 9 and 1 : 4 Sylgard® 184 : FAS ratios provided the ideal particle embedded polymer systems to preserve surface morphology and pore structures (copper 60 mesh substrates), establish preferred wettabilities and achieve surface durability.

The most favorable separation meshes were identified as a result of ensuring surface morphologies and open pore structures were preserved whilst tailoring the surface energy to reside between that of oil and water (approximately between 30 mN m^−1^ and 70 mN m^−1^).^42^ Trends were identified that indicated solvent density had a significant part to play. For example, copper 100 mesh separation rates were substantially higher using toluene–water solutions as opposed to hexane for identical surface coatings. This was attributed to toluene's increased density of 865 kg m^−3^,^43^ as opposed to 672 kg m^−3^ for hexane.^44^ A denser solvent was able to more quickly permeate a mesh substrate when originally mixed with water.

## 4. Conclusion

A facile route to fabricate a superior (super)hydrophobic and oleophilic separation surface was devised throughout this research. Hierarchical surface roughening in combination with silicon-based polymers generated water repellent coatings with oil-loving properties. Numerous different combinations and concentrations of dual scale TiO_2_ and/or hydrophobic-SiO_2_ (H-SiO_2_) particles in varying Sylgard®184 : FAS polymer ratio mixtures were explored in order to enhance surface wettabilities. Average water contact angles were linked to improved solvent separation efficiencies, which, in turn, were most favourable on samples with higher quantities of the fluorine-rich fluoroalkylsilane, FAS, polymer. For example, coating F's dual scale TiO_2_ particle system embedded in a 1 : 4 Sylgard® 184 : FAS polymer mixture was applied to copper 60 mesh substrates (pore diameter of 250 μm). The resulting device was highly functional with 100 ± 0%, 85 ± 0% and 97 ± 0% separation efficiencies for toluene–, hexane– and dichloromethane–water solutions respectively. A trade-off between the highly adhesive and durable Sylgard® 184 polymer and the low surface energy FAS, in a 1 : 4 ratio, afforded robust separation coatings that showed no deviation in functionality after 3 wash–separation–wash cycles nor after the Scotch tape test.

## Conflicts of interest

There are no conflicts to declare.

## Supplementary Material

RA-009-C9RA02700A-s001
