# Supplementary material for: Dual-scale TiO2 and SiO2 particles in combination with a fluoroalkylsilane and polydimethylsiloxane superhydrophobic/superoleophilic coating for efficient solvent–water separation
Source: RSC Adv. 2019 Jun 28;9(35):20332–40. doi: 10.1039/c9ra02700a (PMC9065573; doi:10.1039/c9ra02700a)
Supplement: RA-009-C9RA02700A-s001 [file RA-009-C9RA02700A-s001.pdf]

Electronic Supplementary Material (ESI) for RSC Advances.
This journal is © The Royal Society of Chemistry 2019

# Supporting Information

# Dual-scale $TiO_2$ and $SiO_2$ particles in combination with a fluoroalkylsilane and polydimethylsiloxane superhydrophobic/superoleophilic coating for efficient solvent-water separation.

Frances L. Heale[1], Maud Einhorn[1], Kristopher Page,[1] Ivan P. Parkin[1] and Claire J. Carmalt[1]*

[1]Department of Chemistry, University College London, 20 Gordon Street, London, WC1H 0AJ.

*Corresponding Author at: Department of Chemistry, University College London, 20 Gordon Street, London, WC1H 0AJ. United Kingdom. Tel: +44 20 7679 7528. E-mail address: c.j.carmalt@ucl.ac.uk (C. J. Carmalt).

**Table S1.** Comprehensive list of various oil-water separation coatings comprising dual-scale surface roughening particles and surface energy lowering polymer mixtures. Hydrophobic-$SiO_2$ (H-$SiO_2$) particles were generated by functionalising the as received $SiO_2$ mineral (5.00 g) in a 1H,1H,2H,2H-perfluorooctyltriethoxysilane (FAS) (1.00g)/ethanol (99.00 g) mixture.

| **Particle Loading/g** | | | | **Mixture of Polymer Stock Solutions/g** | | **Sylgard® 184:FAS ratio** |
|---|---|---|---|---|---|---|
| **$TiO_2$** | | **H-$SiO_2$** | | | | |
| **21 nm** | **60-200 nm** | **0.5-1.0 µm** | **5-15 µm** | **Sylgard® 184** | **FAS** | |
| 1.50 | 1.50 | - | - | 30.00 | - | 1:0 |
| 1.50 | 1.50 | - | - | - | 30.00 | 0:1 |
| 1.50 | 1.50 | - | - | 15.00 | 15.00 | 1:1 |
| 1.50 | 1.50 | - | - | 6.00 | 24.00 | 1:4 |
| 1.50 | 1.50 | - | - | 3.00 | 27.00 | 1:9 |
| 0.60 | - | - | 0.60 | 30.00 | 0.00 | 1:0 |
| 0.60 | - | - | 0.60 | 0.00 | 30.00 | 0:1 |
| 0.60 | - | - | 0.60 | 15.00 | 15.00 | 1:1 |
| 0.60 | - | - | 0.60 | 6.00 | 24.00 | 1:4 |
| 0.60 | - | - | 0.60 | 3.00 | 27.00 | 1:9 |
| - | - | 0.60 | 0.60 | 30.00 | - | 1:0 |
| - | - | 0.60 | 0.60 | - | 30.00 | 0:1 |
| - | - | 0.60 | 0.60 | 15.00 | 15.00 | 1:1 |
| - | - | 0.60 | 0.60 | 6.00 | 24.00 | 1:4 |
| - | - | 0.60 | 0.60 | 3.00 | 27.00 | 1:9 |

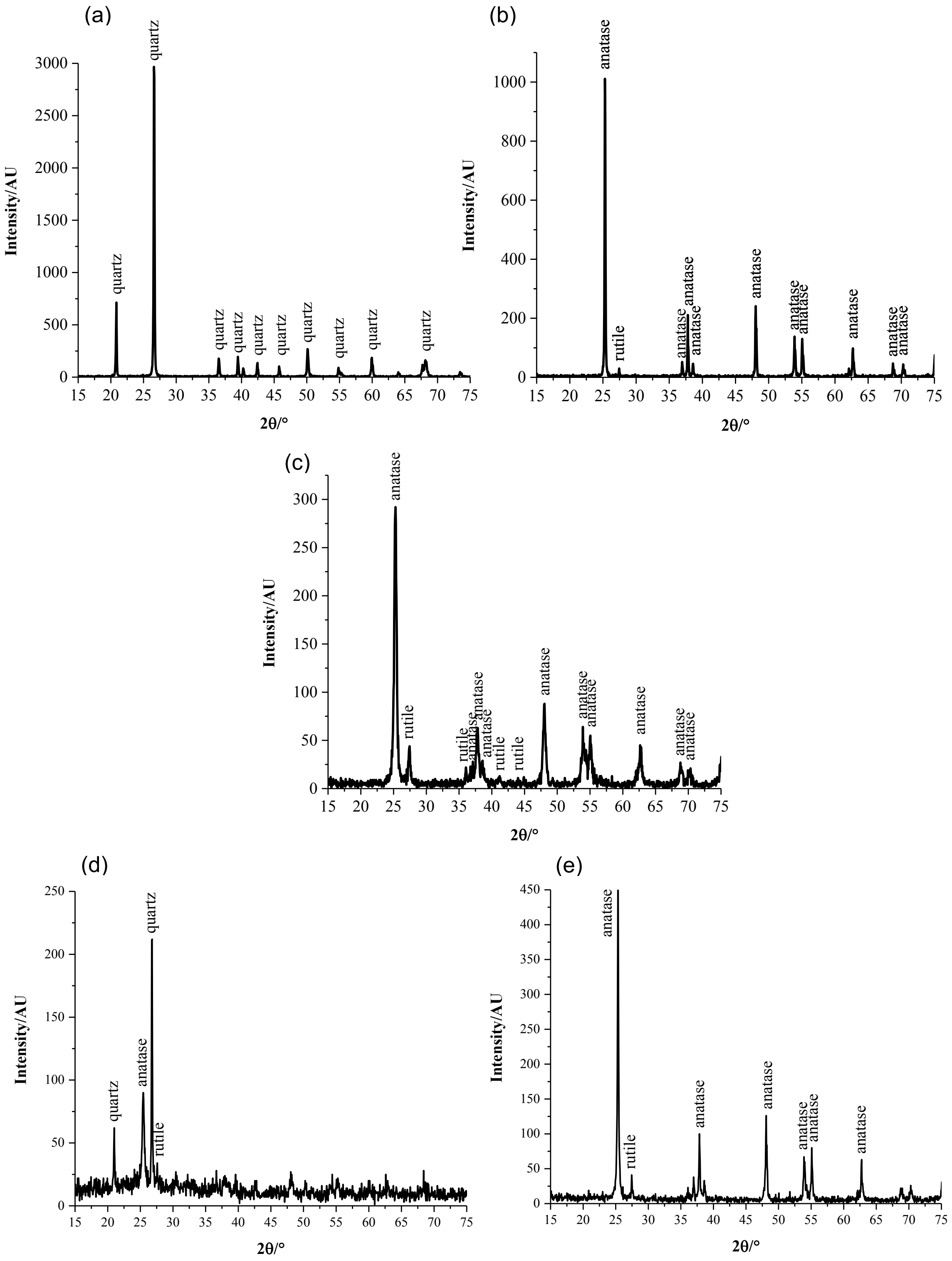


**Figure S1.** X-ray diffraction (XRD) patterns of **(a)** $SiO_2$ 5-15 μm particles, **(b)** $TiO_2$ 60-200 nm particles, **(c)** $TiO_2$ 21 nm particles, **(d)** coating **D** consisting of $SiO_2$ 5-15 μm particles (0.6 g) and $TiO_2$ 21nm particles (0.6 g) in a 1:9 polymer mixture of Sylgard® 184 and FAS and **(e)** coating **F** comprising $TiO_2$ 60-200 nm particles (1.5 g) and $TiO_2$ 21 nm particles (1.5 g) in a 1:1 mixture of Sylgard® 184 and 1H,1H,2H,2H-perfluorooctyltriethoxysilane (FAS).

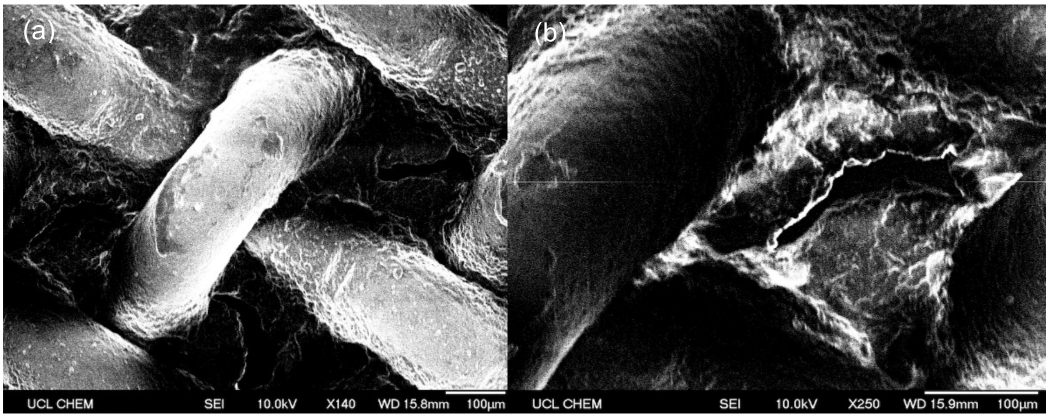


**Figure S2.** Scanning electron microscope (SEM) images of copper 60 mesh substrates coated with 60-200 nm and 21 nm particles $TiO_2$ particles (totalling 3.0 g in equal parts) in **(a)** 1:1 polymer mixture of Sylgard® 184:FAS (30.0 g) and **(b)** 1:0 polymer mixture of Sylgard® 184:FAS (30.0 g). Pores are obstructed in both images but the coating is visibly thicker in **(b)**.

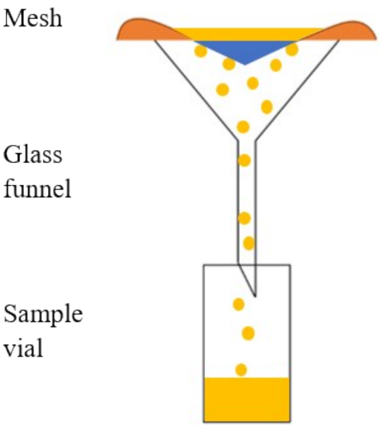


**Figure S3.** Schematic of solvent (yellow) permeating through holes in the functional solvent-water separation device. Water (blue) was unable to flow through mesh pores and therefore collected on the surface. The mass of solvent collected after 30 s of separation provided relevant information for the calculation of a comparable separation efficiency percentage.

**Table S2.** Toluene, hexane and dichloromethane-water separation efficiencies on functionalised 60 and 100 mesh copper substrates. Coating **D** consists of H-$SiO_2$ 5-15 μm particles (0.6 g) with $TiO_2$ 21 nm particles (0.6 g) in a 1:9 mixture of Sylgard® 184 to FAS. Coating **E** comprises of H-$SiO_2$ 5-15 μm particles (0.6 g) with $TiO_2$ 21 nm particles (0.6 g) in a 0:1 mixture of Sylgard® 184 to FAS. Coating **F** consists of $TiO_2$ 60-200 nm particles (1.5 g) and $TiO_2$ 21 nm particles (1.5 g) in a 1:4 mixture of Sylgard® 184 and FAS. Coating **G** contains H-$SiO_2$ 5-15 μm particles (0.6 g) with $TiO_2$ 21 nm particles (0.6 g) in a 1:4 mixture of Sylgard® 184 to FAS.Hydrophobic-$SiO_2$ (H-$SiO_2$) particles were generated by functionalising the as received $SiO_2$ mineral (5.00 g) in a 1H,1H,2H,2H-perfluorooctyltriethoxysilane (FAS) (1.00g)/ethanol (99.00 g) mixture.

| Coating Label | Mesh Pore Size | Solvent Percentage in Solvent-water Mixture Used for Solvent Separation Efficiency Testing/% | | | Solvent Separation Efficiency/% | |
|---|---|---|---|---|---|---|
| | | Toluene-water | Hexane-water | Dichloromethane-water | Average | Error |
| **D** | 60 | 50 | - | - | 100 | 0 |
| **E** | 60 | 50 | - | - | 94 | 2 |
| **F** | 60 | 50 | - | - | 100 | 0 |
| **G** | 60 | 50 | - | - | 95 | 2 |
| **D** | 60 | - | 50 | - | 77 | 3 |
| **E** | 60 | - | 50 | - | 79 | 1 |
| **F** | 60 | - | 50 | - | 85 | 1 |
| **G** | 60 | - | 50 | - | 87 | 0 |
| **D** | 60 | - | - | 50 | 96 | 1 |
| **E** | 60 | - | - | 50 | 59 | 1 |
| **F** | 60 | - | - | 50 | 97 | 1 |
| **G** | 60 | - | - | 50 | 60 | 1 |
| **D** | 100 | 50 | - | - | 93 | 4 |
| **E** | 100 | 50 | - | - | 99 | 1 |
| **F** | 100 | 50 | - | - | 89 | 3 |
| **G** | 100 | 50 | - | - | 96 | 1 |
| **D** | 100 | - | 50 | - | 86 | 3 |
| **E** | 100 | - | 50 | - | 88 | 5 |
| **F** | 100 | - | 50 | - | 78 | 2 |
| **G** | 100 | - | 50 | - | 85 | 2 |
